# A stepwise diagnostic framework for recurrent unexplained fever in adults: an evidence-based review with focus on Latin America

**DOI:** 10.1016/j.clinsp.2026.100992

**Published:** 2026-05-12

**Authors:** Maria Helena Favarato, Victoria Luisa Pereira Aguiar, Edison Ferreira Paiva, Leonardo Oliveira Mendonça

**Affiliations:** a Universidade Municipal de São Caetano do Sul (USCS), São Caetano do Sul, SP, Brazil; b Discipline of Internal Medicine, Faculdade de Medicina da Universidade de São Paulo (FMUSP), São Paulo, SP, Brazil; c Discipline of Clinical Immunology and Allergy, Faculdade de Medicina da Universidade de São Paulo (FMUSP), São Paulo, SP, Brazil

**Keywords:** Fever, Recurrent fever, Review, Recurrent fever of unknown origin, Fever of unknown origin (FUO), Diagnostic algorithm, Tropical fever, Autoinflammatory diseases, Adult infectious diseases, Latin America, Brazil, Clinical decision support, Diagnostic flowchart

## Abstract

**Background and methods:**

Recurrent fever is a complex clinical presentation that poses significant diagnostic challenges, particularly when no clear etiology emerges despite extensive investigation. This integrative narrative review provides a comprehensive overview of recurrent fever and proposes a practical diagnostic flowchart focused on the most frequent etiologies, with particular emphasis on the Latin American and Brazilian contexts. Literature from MEDLINE/PubMed, LILACS, and SciELO was reviewed to summarize evidence on epidemiology, differential diagnosis, and diagnostic strategies.

**Results:**

Recurrent fever may arise from infectious diseases, autoimmune or autoinflammatory disorders, malignancies, metabolic or occupational conditions, drug-induced etiologies, and factitious causes. Infections, notably tuberculosis, malaria, leishmaniasis, and endocarditis, remain leading contributors in developing countries. Non-infectious causes such as rheumatologic conditions, neoplasms, and inborn errors of immunity must also be considered, especially in persistent or unexplained cases. A structured, tiered approach to diagnosis is recommended, beginning with basic laboratory and imaging studies, progressing to targeted invasive procedures and advanced modalities such as 18F-FDG PET/CT, and, when appropriate, genomic sequencing. The flowchart proposed in this review emphasizes clinical history, frequent reassessment, and the judicious use of diagnostic resources to balance cost-effectiveness and diagnostic yield. Moreover, distinguishing between inflammatory and non-inflammatory patterns, as well as identifying the newly described Syndrome of Undifferentiated Recurrent Fever (SURF), may guide further evaluation. Ultimately, recurrent fever requires a systematic and multidisciplinary approach. Incorporating epidemiological context and advances in molecular and imaging technologies may improve diagnostic accuracy and patient outcomes.

## Highlights

• Stepwise diagnostic algorithm for recurrent unexplained fever in adults.
• Integrates infectious, autoimmune, neoplastic, and genetic causes.
• Emphasizes Latin America epidemiology and endemic infections.
• Combines basic tests with PET/CT and genomic sequencing.
• Introduces SURF in persistent unexplained recurrent fever cases.

## Introduction

In 1961, Petersdorf and Beeson^1^ introduced the term Fever of Unknown Origin (FUO), defining it as a temperature exceeding 38.3 °C persisting for more than three weeks, without a definitive diagnosis after one week of inpatient evaluation. With advancements in diagnostic capabilities ‒ even in outpatient settings, alternative definitions have been proposed. One such updated criterion defines FUO as a fever without diagnosis after three days of hospitalization or two outpatient visits.^2^ These evolving definitions highlight that FUO is inherently dependent on context. Diagnostic interpretation is influenced not only by the availability of diagnostic resources but also by the patient’s epidemiological background and the social, cultural, and economic characteristics of the community. Consequently, the diagnostic approach must be guided by the most probable etiologies, which are determined by factors such as disease prevalence, the patient’s country of origin, and their current location.

Similarly, the definition of recurrent fever is shaped by both temporal and geographic factors. One proposed definition describes recurrent fever as episodes of prolonged febrile illness separated by afebrile intervals ranging from 48-hours to several weeks.^3^ The diagnostic workup of recurrent fever often overlaps with that of FUO, as recurrent febrile episodes without an established cause may ultimately fall within the FUO spectrum.

Distinguishing between FUO, recurrent fever, and periodic fever is essential for accurate diagnosis and appropriate clinical management. Equally important is the contextualization of inflammatory fever as a distinct clinical entity. A clear understanding of these classifications enables healthcare professionals to implement targeted diagnostic strategies and therapeutic interventions, ultimately improving patient outcomes.

It is also critical to acknowledge that the interpretation of body temperature and fever can vary depending on factors such as the anatomical site of temperature measurement and the type of device used, both of which may influence the assessment and classification of fever. Despite these potential variabilities, the following paragraphs present a general framework for defining fever and its categorization:

A) Definition of fever in temperature:
a) Low-grade: 37.3 to 38.0 °C (99.1 to 100.4°F);
b) Moderate grade: 38.1 to 39.0 °C (100.6 to 102.2°F);
c) High-grade: 39.1 to 41 °C (102.4 to 105.8°F);
d) Hyperthermia: Greater than 41 °C (105.8°F).
B) Fever of Unknown Origin (FUO): a temperature of 101 ^°^F (38.3 degrees Centigrade) or higher with a minimum duration of three weeks without an established diagnosis after an intensive one-week investigation in the hospital.
C) Periodic fever: Refers to recurring episodes of illness in which fever (higher than 37.3 degrees centigrade) is the cardinal feature, episodes have a predictable symptom complex and duration, and episodes are separated by symptom-free intervals.
D) Recurrent fever: Is when the patient is in a febrile (higher than 37.3 degrees centigrade) period for a few days and then follows a period of another few days without fever (afebrile), and then the situation repeats, with a few days with fever and then a few afebrile days.
E) Recurrent unexplained fever: Recurrent fever refers to febrile episodes lasting several days, followed by afebrile intervals of similar duration, with this pattern repeating over time.
F) Inflammatory fever: Inflammatory fever is defined as fever accompanied by elevated inflammatory markers, indicating systemic inflammation, without a clear focus or with a degree of inflammation disproportionate to the identified infectious, allergic, or neoplastic process.
G) Syndrome of Undifferentiated Recurrent Fever (SURF): Refers to recurrent or periodic fever without a definitive diagnosis, despite adequate investigation, and with evidence of an inflammatory basis.^4^

A comprehensive understanding of the most prevalent etiologies is essential for constructing a rational and efficient diagnostic algorithm. However, there is a notable paucity of studies characterizing the current epidemiological landscape of recurrent fevers in adults in Latin America, particularly in Brazil.

## Objectives

This integrative review aims to summarize the main causes of recurrent fever and to propose a diagnostic flowchart aligned with the most frequent etiologies, with a particular focus on Latin America and Brazil. Fever of unknown origin related to healthcare-associated settings or immunocompromised states is beyond the scope of this review.

## Methods

A non-systematic narrative overview^5^ was conducted with the objective of pragmatically synthesizing the available scientific literature on the clinical investigation of recurrent fever, with particular emphasis on its main etiologies and their relevance in the Brazilian and Latin American context. In accordance with recommendations for narrative reviews, the search strategy was intentionally broad, allowing inclusion of diverse study designs and sources. Literature search was performed in multiple electronic databases, including MEDLINE/PubMed, LILACS, and SciELO, to capture both international and regional publications. In addition, complementary manual searches were conducted by screening the reference lists of key articles, relevant reviews, and national clinical guidelines, as well as consulting gray literature sources such as institutional reports, theses, and official documents when pertinent. Search terms were selected based on Medical Subject Headings (MeSH) and DeCS descriptors such as ‘fever’, ‘recurrent fever’, ‘fever of unknown origin’, as well as free-text keywords related to recurrent fever, periodic fever syndromes, diagnostic approaches, and infectious, inflammatory, and autoinflammatory conditions, combined using Boolean operators (AND/OR) to maximize retrieval.

The article selection process prioritized high-quality primary studies, narrative and systematic reviews, case series, and clinical guidelines that provided clinically relevant information and broadly reflected the Brazilian epidemiological and healthcare context. No strict restrictions regarding study design or publication date were applied, in line with the exploratory goals, although emphasis was placed on publications with clear methodological descriptions and clinical applicability. Articles in English, Portuguese, and Spanish were considered.

Ethical review board approval was not required for this study, as it exclusively involved the analysis and synthesis of previously published data. No human participants were directly involved, and no personal, sensitive, or identifiable data were collected or analyzed. Consequently, the study did not include any procedures that would require ethical approval under current national or international guidelines for research involving human subjects.

To facilitate interpretation and provide a practical overview of the most relevant evidence, a summary table of the key studies and clinical guidelines referenced in this narrative review is presented (Table 1). This table highlights study design, population, main findings, and clinical relevance, particularly in the Brazilian context. The table summarizes key studies and reviews that have shaped the diagnostic approach to recurrent and prolonged fever, spanning foundational concepts, structured diagnostic strategies, and emerging etiologies. It does not aim to be exhaustive but rather to summarize representative studies and guidelines that underpin the main diagnostic and clinical considerations discussed throughout the text.

**Table 1:** Key studies and guidelines underpinning the diagnostic approach to recurrent and prolonged fever.

Author / Year	**Country**	**Study type**	**Population / Scope**	**Main focus**	**Key findings / Clinical relevance**
Petersdorf & Beeson, 1961	USA	Case series	100 patients with prolonged fever	Original description of FUO	Seminal work defining fever of unknown origin and establishing the foundation for subsequent diagnostic frameworks.
Reimann & McCloskey, 1974	USA	Narrative review	Patients with periodic fever	Periodic fever syndromes	Early recognition of recurrent/periodic fever as a distinct clinical problem, emphasizing diagnostic complexity and longitudinal assessment.
Durack & Street, 1991	USA	Narrative review / Conceptual framework	Patients with FUO	Redefinition of FUO	Updated FUO definitions and diagnostic categories, widely adopted in clinical practice and research.
Knockaert et al., 1992	Belgium	Observational study	Adults with FUO	Etiological spectrum	Demonstrated changes in FUO causes over time, reinforcing the need for updated diagnostic strategies.
Knockaert et al., 1993	Belgium	Case series and literature review	Recurrent or episodic FUO	Recurrent FUO	Characterized recurrent/episodic FUO as distinct from continuous FUO, with implications for follow-up and investigation.
Bleeker-Rovers et al., 2007	Netherlands	Prospective multicenter study	Adults with FUO	Structured diagnostic protocol	Showed that a stepwise, protocol-driven approach significantly increases diagnostic yield in FUO.
Vanderschueren et al., 2003	Belgium	Observational cohort	Adults with prolonged fever	Prolonged fever vs. FUO	Highlighted the transition from prolonged febrile illness to FUO and persistent diagnostic challenges.
Papa et al., 2021	Italy	Observational study / Expert review	Patients with recurrent fever	SURF	Defined Syndrome of Undifferentiated Recurrent Fever (SURF) as an emerging autoinflammatory condition relevant to unexplained recurrent fever.
Sutera et al., 2022	Italy	Observational cohort	Patients with SURF	Long-term outcomes	Provided clinical characterization, follow-up data, and treatment responses in SURF patients.
Zenone, 2015	France	Narrative review	Adults with recurrent prolonged fever	Diagnostic approach	Proposed a pragmatic, etiology-oriented diagnostic algorithm for recurrent fever in adults.
Betrains et al., 2022	International	Systematic review and meta-analysis	FUO patients	Rheumatic causes of FUO	Quantified the contribution of rheumatic diseases to FUO, supporting systematic inflammatory evaluation.

## Results and discussion

### Epidemiological aspects

The prevalence of unexplained fever is estimated to be approximately 45 % in community hospitals[6] and ranges from 18 % to 50 % of Fever of Unknown Origin (FUO) cases in tertiary care centers.^7^^,^^8^ It is more frequently observed among patients who receive a delayed diagnosis or remain without a definitive diagnosis despite extensive evaluation,^9^ highlighting the increasing diagnostic complexity encountered across different levels of the healthcare system. Infections remain the most frequent cause of fever of unknown origin in developing countries[10] and, in a Brazilian case series,^11^ infectious diseases accounted for 43 % of FUO cases, with tuberculosis identified as one of the most frequent etiologies. In a single-center Egyptian cohort, a continuous pattern of fever was found in 58.3 % of patients, with 23.2 % with intermittent fever symptoms, and 1.6 % had relapsing fever.^10^

Compared to other FUO patients, individuals with recurrent fever are generally younger and exhibit a longer duration of symptoms, with approximately 50 % experiencing symptoms for more than one year. The etiologies of recurrent fever can be broadly categorized into infectious diseases, malignancies, immune-mediated rheumatologic conditions, and autoinflammatory disorders. Notably, the proportion of patients who remain without a definitive diagnosis ranges from 42 % to 53 %.^12^ Moreover, this subgroup is more likely to remain undiagnosed at the conclusion of the diagnostic workup.^12^ Recurrent fever is a challenge even if genetic tests directed to monogenic recurrent fevers are available.^13^^,^^14^

Recurrent fever is most commonly due to infectious diseases ‒ malaria, rickettsioses, relapsing fever borreliosis, enteric fever, and leishmaniasis ‒ whose relative frequencies depend on geography and patient population. Non-infectious causes are less common but should be considered when infectious etiologies are excluded. A significant fraction of cases remains undiagnosed, underscoring the need for improved diagnostics and standardized study designs.^15^, ^16^, ^17^, ^18^

### Diagnostic investigation

**Fig. 1:**
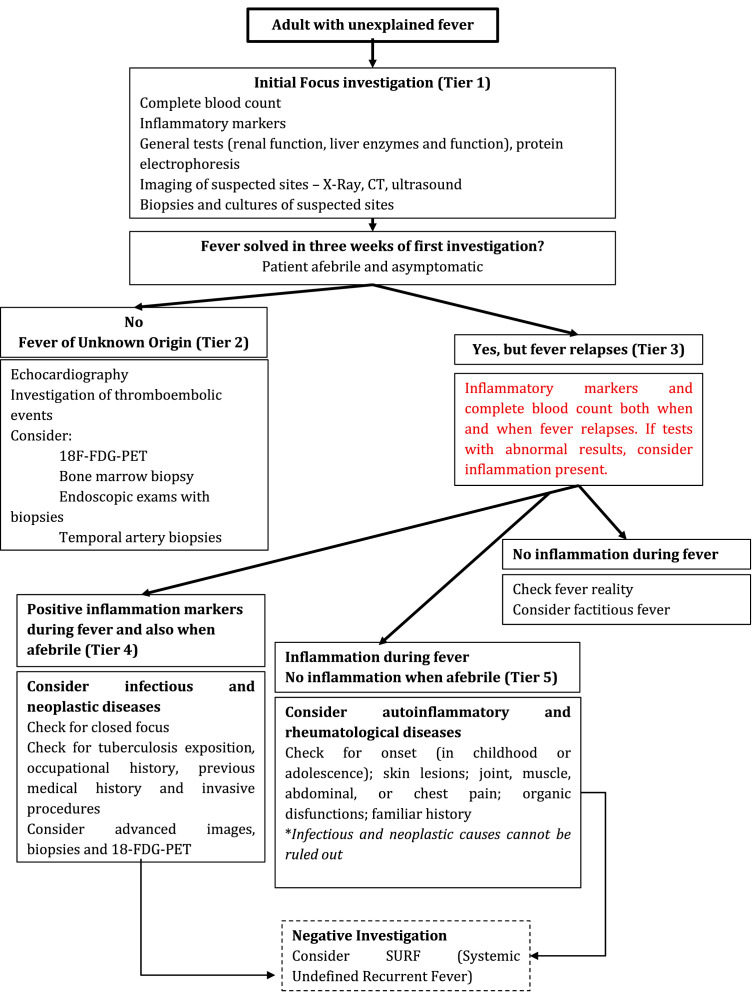
Proposed flowchart of investigation.

A major pitfall in diagnostic investigation is, as previously mentioned, that there is not enough epidemiological information to reliably know the probability of each of the possible causes of recurrent fever in adults in real clinical practice scenarios. The investigation should be based on a thorough patient history and complemented by epidemiological risk factors, such as travel history, occupation, endemic area, and past medical history, which are initial decision points in the diagnostic path. Next, proceed to a detailed and extensive physical examination. Clinical history, disease progression, and pathological findings are considered the most informative elements for establishing a diagnosis, with clinical evaluation being the primary driver of early diagnostic suspicion.^9^ On the other hand, caution should be exercised with an excess of questions and the way they are asked, as they can lead to incorrect paths, resulting in inconclusive outcomes and futile investigations.^6^ Furthermore, careful observation of progression also increases the possibility of establishing a diagnosis.^9^ Therefore, the authors suggest frequent clinical evaluation of the patient with suspected fever without a clear focus to both categorize the febrile episodes and to guide subsequent laboratory/imaging investigation. A practical flowchart (Fig. 1) as a guide for the investigation of fever without a clear focus is proposed.

Nonspecific laboratory analysis may offer useful clues for unexplained fever, and the sensitivity of these exams grows when they are used combined. However, excessive investigation may not just be not necessary and costly but also may induce a patient's trauma. To this, the authors propose a stepwise approach for laboratory, imaging and invasive studies investigation to guide general physicians, presented both in Fig. 1 and complemented with Table 2. Tier 1 is suggested as the initial investigation over the course of the first three weeks of an unexplained fever (Table 2). Tier 2 is suggested for the scenario where an unexplained fever lasts for more than three weeks, and then hospitalization is suggested for clinical investigations. Tier 3 is suggested after fever resolution, particularly in patients who subsequently experience relapse. This aims to first check laboratory normalization (focusing on classical inflammatory markers and blood count) and to guide patient follow-up. Therefore, if laboratory abnormalities persist after fever resolution, at least one additional follow-up evaluation (Tier 4) is recommended, consisting at least of the inflammatory markers and repetition of other tests directed by clinical and previous laboratory findings. Tier 5 is suggested for situations of high complexity, especially due to the use of medical genomics.

**Table 2:** Suggested tiers for recurrent fever stepwise investigation.

	**Tier 1**	**Tier 2**	**Tier 3**	**Tier 4**	**Tier 5**
**Suggestion**	Initial investigation over the course of the first three weeks	Fever lasts for more than three weeks. Suggested hospitalization for clinical investigation	Investigation both febrile and afebrile when fever relapses.	Laboratory aberrations persist after fever cessation	High-complexity situations
**Laboratory**	Complete blood count; Inflammatory markers (CRP, ESR and ferritin); General tests (renal function, liver enzymes, protein electrophoresis and hepatic evaluation) Microbiological and serological tests (smear tests and cultures from all suspected sites)	Guided by symptoms and epidemiological data	Repeat inflammatory markers and complete blood count	Follow up to confirm fever relapses and new laboratory analysis in 2-weeks	Genomic testing
**Imaging**	Imaging of suspected sites: X-Ray, CT or ultrasound.	Echocardiography; Investigation of thromboembolic events; Consider the combination of positron emission tomography with 18F-fluorodeoxyglucose (18F-FDG-PET) and computed tomography		Consider imaging for closed focus infections (spine, intra-abdominal or pelvic abscesses)	
**Invasive exams**	Biopsies and culture of suspected sites	Bone marrow biopsy; Endoscopic exams with biopsies; Temporal artery biopsy		Biopsies and culture of suspected sites	

In Tier 1, during the first three weeks of evaluation in the patient with fever of unknown origin, the authors suggest that these patients may have at least once: (1) A complete blood count; (2) Inflammatory markers (CRP, ESR and ferritin); (3) And general tests such as renal function, liver enzymes, protein electrophoresis, and hepatic evaluation. The authors also suggest that smear tests and cultures from all suspected sites should be done, upon demand. Imaging suspected sites may also be requested, but may be limited to X-Ray, CT, or ultrasound.

If fever persists over 3-weeks of duration, the authors suggest evaluation as Fever of Unknown Origin – FUO ‒ considering hospitalization for extensive laboratory/imaging investigation. Further tests, Tier 2, should be guided by symptoms and epidemiological data and may include echocardiography (both transthoracic and transesophageal), bone marrow biopsy, endoscopic exams with biopsies, temporal artery biopsy, and investigation of thromboembolic events.^12^ The combination of Positron Emission Tomography with 18F-Fluorodeoxyglucose (18F-FDG-PET) and computed tomography is considered a promising diagnostic tool in the investigation of fever or inflammation of unknown origin, as it combines high spatial resolution with the detection of increased glycolysis due to malignancy or inflammation. However, in a prospective study, 30 % of the scans were “false positives”, with increased diagnostic prediction when more nonspecific criteria were excluded to consider the scan positive. Nonetheless, the study concluded that the performance of 18F-FDG-PET in investigating fever or inflammation of unknown origin had a specificity of 53.8 %, sensitivity of 91.8 %, positive predictive value of 76 %, negative predictive value of 80.6 %, and diagnostic accuracy of 77.1 %.^19^ Thus, PET imaging should be considered as a second-line imaging test in the investigation of patients with a fever of unknown origin.^20^^,^^21^

Indeed, genomic medicine should also be considered, Tier 5, to investigate both persistent fever and recurrent unexplained fever. The use of metagenomics for the investigation of specific and broad infective agents has been revised by Dong et al., suggesting that the use of quantitative metagenomics next-generation sequencing can be costly and time-consuming.^22^ Moreover, targeted gene panels or unbiased exome/genome sequencing may be useful for identifying underlying immunological or metabolic disorders. These tests should be considered in cases of unexplained or recurrent sterile fever, atypical infections, or overlapping autoimmune, infectious, and neoplastic features. One may be aware of the limitations of each type of genetic sequencing, especially for the investigation of adult patients. The use of genome sequencing in a single tertiary center investigation, in patients with unexplained fever syndrome, resulted in a negative 80 % of the time, but several considerations must be taken to correctly interpret this negativity.^23^

A fully investigated patient with unexplained fever remains a concerning case. Encountering a patient who has undergone a comprehensive evaluation yet continues to experience fever presents an even greater challenge. The authors recommend considering the distinction between inflammatory and non-inflammatory recurrent fever by measuring inflammatory markers during and outside of febrile episodes. This approach aims to identify autoinflammatory or immune dysregulatory syndromes, for which the term Syndrome of Undifferentiated Recurrent Fever (SURF) has been proposed. This concept is important for guiding the physician in lines of investigation, considering the use of genetic resequencing, and identifying other immunological assessments that may be warranted over the course of follow-up.

Therapeutic trials, especially with glucocorticoids, antibiotics and colchicine, should be avoided whenever the patient is clinically stable, as they may delay the diagnostic process.^24^

When initial investigations fail to clarify the diagnosis and the fever remains unexplained, referral to a reference center or tertiary hospital may improve diagnostic and therapeutic outcomes.^25^ Referral for specialist evaluation is indicated when the fever persists despite a detailed initial clinical assessment and basic laboratory investigation, or when there are concerning features such as prolonged duration (> 2‒3 weeks), high fever (> 38.3 °C), or associated symptoms suggestive of serious underlying disease, such as weight loss, lymphadenopathy, splenomegaly, arthralgia, or cutaneous manifestations.^25^, ^26^, ^27^, ^28^

Patients with recurrent fever and signs of immunodeficiency, suspected autoinflammatory syndromes, malignancy, or unexplained laboratory abnormalities (e.g., cytopenias, persistent elevation of inflammatory markers) should be referred for evaluation by infectious diseases, immunology, rheumatology, or hematology, depending on the clinical context.^29^

In the next session, the main etiologies of recurrent fever are presented in groups, exploring epidemiological, clinical, and complementary exams related to each disease or group of diseases.

### Recurrent fever etiological groups

#### Infectious causes

Relapsing fever of infectious origin should raise suspicion for infections caused by slow-growing pathogens ‒ such as mycobacteria and certain fungi ‒ as well as infections localized in closed anatomical sites. In a Brazilian case series,^11^ infectious diseases accounted for 43 % of Fever of Unknown Origin (FUO) cases, with tuberculosis identified as one of the most frequent etiologies. Tuberculosis continues to pose a significant clinical challenge, particularly in developing countries, and has been designated a global public health emergency by the World Health Organization since 1993.^30^ Among the 30 countries with the highest reported incidence of tuberculosis between 2019 and 2021, Brazil ranks eighteenth ‒ the only Latin American country on the list ‒ and has experienced an increase in reported cases during this period.^30^ Brazil also reports the highest rate of tuberculosis and HIV co-infection globally.^30^^,^^31^ Tuberculosis may manifest as recurrent fever, especially in its extrapulmonary forms and in patients with comorbidities such as renal or hepatic insufficiency. A notable feature is the protracted course prior to diagnosis, which may extend up to three years.^32^

Additional infectious causes of relapsing or persistent fever include endocarditis ‒ particularly relevant in Brazil due to the high prevalence of rheumatic heart disease^33^ ‒ as well as malaria, abscesses, toxoplasmosis, salmonellosis, and schistosomiasis.

In tropical and subtropical regions of Latin America, several areas remain endemic for malaria, with most cases in South America concentrated within the Amazon basin. In 2015, four countries accounted for approximately 83 % of malaria cases in the Americas: Brazil (24 %), the Bolivarian Republic of Venezuela (30 %), Colombia (10 %), and Peru (19 %).^34^

Tick-borne diseases in the region involve complex epidemiological and ecological interactions between vectors, pathogens, and a variety of host species.^35^ Within the *Borrelia* genus, species responsible for relapsing fever are distinct from those causing Lyme borreliosis, now classified under the genus *Borreliella*. In Latin America, the relapsing fever group of *Borrelia* remains insufficiently characterized, with the vectors for certain species still unidentified. Taxonomic and genetic studies are needed to determine which species of soft ticks may serve as vectors for these pathogens.^36^ In Brazil, such infections are not consistently reported and are rarely included in the differential diagnosis of febrile illnesses. Nevertheless, recent findings have documented the isolation of *Borrelia* species potentially pathogenic to humans in the country.^37^

Regarding Lyme disease, countries along the cooler, high-altitude regions of the Andes Mountains are more susceptible. Although no confirmed cases have been reported in peer-reviewed literature or official medical records since the early 20th century, its presence cannot be entirely excluded, particularly among indigenous populations in mountainous environments.^36^ In Brazil, serological findings and cutaneous lesions resembling erythema migrans have been reported; however, no *Borrelia* species has been definitively associated with these cases,^35^ and *Borrelia burgdorferi* has never been isolated in the country. Baggio-Yoshinari Syndrome is an emerging tick-borne disease in Brazil that clinically mimics Lyme disease. While classical Lyme disease is caused by spirochetes of the *Borrelia burgdorferi* sensu lato complex and transmitted by Ixodes ticks (primarily from the Ixodes ricinus complex), BYS is associated with hard ticks of the genera Amblyomma, Rhipicephalus, and Dermacentor. The syndrome is characterized by erythema migrans-like skin lesions, flu-like symptoms, and arthritis following a tick bite, and cases of prolonged symptoms suggestive of latent infection have been reported.^38^ Despite these clinical similarities, no specific *Borrelia* species has been isolated from Baggio-Yoshinari Syndrome patients, and further studies are needed to investigate potential immunogenetic predispositions in seronegative individuals.

Visceral leishmaniasis is a recognized cause of prolonged and recurrent fever. Brazil accounts for 97 % of cases in America.^39^

Other infectious etiologies that should be considered include localized bacterial foci, such as infections of the biliary tract, sigmoid colon, urinary tract (including prostatitis), dental abscesses, and deep-seated sites such as the mastoid, retroperitoneum, or bone (osteomyelitis). Additionally, infections associated with foreign materials ‒ such as endovascular prostheses, orthopedic implants, and central venous catheters ‒ are important potential sources of fever.^12^ Accordingly, imaging studies play a critical role in the diagnostic workup, alongside the collection of appropriate culture samples.

Patients presenting with unexplained fever may also include returning travelers. While most illnesses in this group are due to common infections with typical clinical presentations ‒ such as acute diarrhea, pneumonia, or pyelonephritis ‒ it is imperative to identify infections that are treatable, potentially life-threatening, or transmissible. Febrile illnesses are a significant concern among travelers returning from tropical and subtropical regions, particularly South America and Southeast Asia. Malaria remains the foremost diagnosis that must be excluded.

Accurate assessment of travel history ‒ including geographic areas visited, activities undertaken, types of accommodations, and the interval between potential exposure and symptom onset ‒ is essential for narrowing the differential diagnosis.^40^ In Brazil, infectious diseases of concern for travelers include enteric infections, typhoid fever, endemic mycoses (such as *Coccidioides spp*., *Cryptococcus neoformans, Histoplasma spp*., and *Paracoccidioides spp*.), influenza, tuberculosis, HIV, other sexually transmitted infections, leptospirosis, schistosomiasis, chikungunya, Zika virus, and dengue fever.^40^ Microbiological and serological tests, such as smear tests and cultures from all suspected sites, should be performed, and biopsies and cultures should be considered. If infective endocarditis is suspected, along with blood cultures, transthoracic/transesophageal echocardiography are needed and one can use other techniques either for the diagnosis of cardiac involvement (cardiac CT, [18F]FDG-PET/CT, or WBC SPECT/CT), or for the diagnosis of distant lesions (cerebral MRI, whole-body CT, and/or PET/CT).^41^

Cyclic neutropenia is characterized by regular episodes of neutropenia, usually every 21-days, during which there is a significant drop in neutrophils, predisposing the patient to bacterial infections and inflammation. During these periods, recurrent fever is common, often accompanied by oral ulcerations, pharyngitis, gingivitis, lymphadenopathy, and skin or respiratory tract infections. Between episodes, the patient tends to remain asymptomatic. The pattern of recurrent fever with a fixed periodicity (approximately 21-days) is a classic finding and can be an important clinical sign for differential diagnosis. Diagnosis must be confirmed by serial blood counts, showing neutrophil fluctuations, and, when indicated, by genetic analysis of the ELANE gene.^42^, ^43^, ^44^

#### Fever related to neoplasms

Fever of Unknown Origin (FUO) attributable to malignancy has an estimated prevalence of 15 %–20 %.^45^ Neoplastic conditions most frequently associated with fever include Hodgkin and non-Hodgkin lymphomas, soft tissue sarcomas, acute and chronic leukemias, and renal cell carcinoma. However, fever can be induced by a broad spectrum of malignancies and even by certain benign tumors, such as atrial myxomas. The underlying pathophysiological mechanisms are not yet fully elucidated.

It is hypothesized that tumor-related fever involves the production of endogenous pyrogens. This has been demonstrated in vitro and supported in vivo by the detection of pyrogenic substances in tumor secretions and tissues from febrile patients with malignancies. Tumor cells are known to secrete pro-inflammatory cytokines that contribute to the febrile response. These pyrogens act on the preoptic area of the anterior hypothalamus, stimulating the synthesis of prostaglandin E2, which subsequently elevates the hypothalamic set point and leads to an increase in body temperature.

Possible mechanisms include the release of these mediators in response to tumor necrosis, bone marrow necrosis, or other yet unidentified pathways. Despite these observations, a comprehensive and definitive explanation for tumor-related fever remains lacking. The overlap in cytokine profiles between infectious and neoplastic processes poses a significant diagnostic challenge. As a result, tumor-related fever must be carefully differentiated from infectious etiologies ‒ whether viral, bacterial, or fungal ‒ as well as from non-infectious causes such as thromboembolic events, tumor lysis syndrome, and nonbacterial thrombotic (marantic) endocarditis.^46^

An additional factor that complicates the diagnostic evaluation of Fever of Unknown Origin (FUO) is the possibility of drug-induced fever. Numerous pharmacologic agents have been implicated, including chemotherapeutic drugs such as bleomycin, daunorubicin, cisplatin, asparaginase, and interferons (20), as well as anticonvulsants, antimicrobials, bisphosphonates, antithymocyte globulin, and monoclonal antibodies.^46^

Beyond pharmacologic causes, several non-infectious and paraneoplastic mechanisms must also be considered. These include transfusion-related febrile reactions, central nervous system metastases involving the meninges or hypothalamus, radiation-induced pneumonitis or pericarditis, and adrenal insufficiency due to either corticosteroid withdrawal or adrenal metastases.^45^ These factors further underscore the complexity of FUO in oncologic and immunocompromised patients, necessitating a broad and systematic diagnostic approach.

A favorable response to anti-inflammatory medications, characterized by the naproxen test ‒ administration of 250 mg orally twice a day, with fever remission within 24-hours and sustained remission if naproxen is continued ‒ forms part of the proposed diagnostic criteria for tumor-related fever. These criteria include:[45]

(I) Temperature above 37.8 °C at least once per day.
(II) Fever lasts >2-weeks.
(III) Absence of evidence of infection in: (A) Physical examination; (B) Laboratory tests, such as smears or cultures of sputum, blood, urine, stool, bone marrow, cerebrospinal fluid, pleural fluid, and local lesion discharge; (C) Imaging studies, such as chest X-Ray and CT scans of the head, abdomen, and pelvis.
(IV) Absence of allergic mechanisms, such as drug allergies, transfusion reactions, radiation, or reactions to chemotherapy drugs.
(V) Lack of fever response to appropriate empirical antibiotic therapy for at least 7-days.
(VI) Immediate and complete cessation of fever with naproxen, with sustained normal temperature while on naproxen.

Cancer of an undetermined site is a diagnostic challenge. A careful history, in-depth clinical examination, and complementary tests targeting the most common sites according to the patient's epidemiological characteristics should be performed. Imaging and PET scans may be useful, as well as biopsies of suspicious sites with histological and immunohistochemical analysis.^47^

#### Fever related to rheumatic diseases

In patients with undiagnosed fever, systemic immune-mediated diseases should be considered, especially when the fever is not very pronounced.^48^ Fever attributed to disease activity occurs in 50 % of patients with systemic lupus erythematosus, for example. Clinical situations in which patients with known rheumatological diseases, with or without ongoing pharmacological treatment, present with fever should also be considered, as it may signify opportunistic infections or drug-induced fever. Another important point is the potential for infectious diseases to present with skin and joint manifestations that can mimic systemic autoimmune diseases, such as viral hepatitis, arboviral diseases, and infection with parvovirus B19.^24^^,^^48^

Rheumatological conditions that may be associated with fever include adult-onset Still's disease; rheumatoid arthritis; connective tissue diseases, especially systemic lupus erythematosus; spondylarthritis; vasculitis such as giant cell arteritis, polyarteritis nodosa, and ANCA-associated vasculitis; other systemic diseases such as sarcoidosis; and crystal-induced arthropathies.^49^ In a recent case series, the most common rheumatological causes of fever were adult-onset Still’s disease, large vessel vasculitis, and systemic lupus erythematosus.^50^

Adult-Onset Still's Disease (AOSD) is a rare and complex systemic inflammatory disorder of unknown etiology characterized by recurrent high fever, evanescent rash, arthralgia or polyarthritis, and leukocytosis with neutrophilia.^51^, ^52^, ^53^ The pathogenesis of AOSD involves a cytokine storm, with excessive production of Interleukins (IL)-1, IL-6, IL-18, Tumor Necrosis Factor-α (TNF-α), and Interferon-γ (IFN-γ), which play a crucial role in disease progression. Clinically, AOSD can manifest in three main patterns: self-limited or monophasic, intermittent or polycyclic, and chronic articular, each with different prognoses.^51^ Diagnosis is often based on the Yamaguchi criteria, which are widely used after exclusion of other infectious, rheumatic, and neoplastic diseases. AOSD can be complicated by serious conditions, such as macrophage activation syndrome, which requires aggressive immunosuppressive treatment.^54^ In addition, the disease can manifest in elderly patients, presenting with different clinical features and complications, such as pleuritis and disseminated intravascular coagulation.^55^^,^^56^

#### Fever related to the inborn errors of immunity

Inborn Errors of Immunity (IEIs) are caused by germline or somatic mutations in single genes and may present with a broad spectrum of clinical manifestations, including increased susceptibility to infections, autoimmunity, hyperinflammation, allergic disorders, bone marrow failure, and/or malignancy. Although fever is a common symptom in many disease processes, the occurrence of recurrent fever ‒ particularly when unexplained ‒ should prompt a thorough immunological evaluation.^57^

This diagnostic consideration has become increasingly relevant following the identification of the molecular mechanisms underlying autoinflammatory diseases. While “sterile pyrexia” is a hallmark feature of autoinflammatory conditions, it is an immunologically mediated phenomenon that may also be observed in other pathological contexts.^58^ It is critical to recognize the immune dysregulation inherent in a range of complex disorders in which recurrent fever may coexist with infectious episodes, rheumatologic features, neoplasia, or persist as fever of unknown origin.

Such disorders are collectively categorized as Primary Immune Regulatory Disorders (PIRDs), a heterogeneous group of conditions characterized by impaired homeostatic control of inflammation and a breakdown in immune tolerance.^59^ The identification of these entities is essential for accurate diagnosis, targeted therapy, and long-term management. The IEI 2024 classification (download available at: https://iuis.org/committees/iei/) lists the PIRD in: (A) T-regulatory cells defects; (B) autoimmunity with or without lymphoproliferation; (C) Immune Dysregulation with colitis; (D) Autoimmune Lymphoproliferative Syndrome (ALPS); (E) Susceptibility to EBV and lymphoproliferative conditions; (F) Autoinflammatory diseases; and (G) Complement deficiencies. Regarding clinical presentation, several features may be suggestive of an underlying inborn error of immunity.^60^ These include early or childhood onset of symptoms; fever that is either moderate or overshadowed by more prominent manifestations; absence of clinical signs between febrile episodes; and systemic findings such as ocular involvement (e.g., conjunctivitis, episcleritis, uveitis, periorbital edema), urticarial or migratory rash, neurological manifestations (including sensory deficits and aseptic meningitis), arthritis, chest or abdominal pain, gastrointestinal disturbances (including diarrhea), myalgia, lymphadenopathy (painful or painless), aphthous ulcers, pharyngitis, and episodes triggered by cold exposure or emotional stress. Additional indicators may include skeletal abnormalities and a positive family history of related symptoms or immune disorders.

During diagnostic evaluation, it is essential to perform a detailed characterization of the symptomatology, including onset, duration, frequency, and periodicity of episodes, along with associated clinical signs as described above. A crucial component of clinical assessment is the exploration of possible patterns of genetic inheritance. This involves obtaining detailed family history extending over at least three generations, which may reveal inheritance trends suggestive of a monogenic immune dysregulation.

Furthermore, the age at symptom onset should be carefully evaluated, as some IEIs ‒ particularly those associated with somatic mutations ‒ may manifest during adulthood, challenging the conventional perception of IEIs as exclusively pediatric conditions.^61^^,^^62^

It is considered that a significant percentage of patients with recurrent fevers do not carry variants of genes associated with monogenic conditions and do not fulfill any diagnostic or classification criteria for PFAPA or other inherited recurrent fever syndromes. Even when genetic testing is available, up to 40 % of patients have no molecular diagnosis. Those could be classified as SURF, Syndrome of Undifferentiated Recurrent Fever,^13^^,^^14^ a heterogeneous group of considered autoinflammatory diseases characterized by self-limiting (median of 4-days) episodes of systemic inflammation without a confirmed molecular diagnosis.^4^ In a cohort that followed patients with SURF for at least one year (median 3.3-years),^13^ 7.8 % were excluded because, in evolution, they showed signs of other chronic diseases that allowed a final diagnosis. Considering SURF, 68 % of patients were men, with a median age at onset of 3-years and a median diagnostic delay of 3.2-years. The median number of episodes per year was 12, characterized by fever (100 %), abdominal pain (62 %), arthralgia and/or myalgia (48 %). Tonsillitis was also a remark, with 38 % with erythematous tonsillitis, 38 % with exudative tonsillitis, and 32 % aphthous stomatitis. Those oropharyngeal manifestations are not always consistent over time, sometimes isolated or observed only sporadically, differentiating those patients from PFAPA syndrome. Almost 65 % achieved a complete and persistent response with colchicine, which was also responsible for 18 % of partial response.^13^ On-demand steroids and on-demand NSAIDs, as well as long-term use of colchicine and interleukin-1 inhibitor anakinra, were the most common therapeutic approaches in the clinical studies addressing SURF.^14^

#### Fever related to metabolic diseases

Metabolic disorders may also result in recurrent fever. In storage diseases such as Fabry disease, fever can also be an initial manifestation, especially in childhood and adolescence. However, it is often underdiagnosed or attributed to other causes, which contributes to significant diagnostic delays. Fabry's disease is caused by lipid accumulation in organs due to the defect of α‐Gal A, causing metabolic disturbances. In the early phase of the disease, the main manifestations are acroparesthesia/acrodynia, hypohidrosis/anhidrosis, and angiokeratomas. Sensorineural hearing loss and nonspecific gastrointestinal symptoms may also appear. The pain crises in these patients are usually associated with fever. In the absence of universal guidelines, the diagnosis is mainly based on clinical manifestations and confirmed by pathological examination, α‐Gal A activity detection, and the *GLA* gene testing. Fever tends to be episodic and may be accompanied by a slight elevation of inflammatory markers.^63^, ^64^, ^65^

Gaucher (type 1) disease may also arise from unexplained fever, as the disease course is characterized by recurrent transient attacks of fever, severe constitutional symptoms associated with amyloidosis, nephropathy, and hyperglobulinemia. The disease may also be associated with neurologic features (types 2 and type 3) with early onset in life. The diagnosis is suggestive in patients with 0 %‒15 % of normal glucocerebrosidase enzyme activity in peripheral blood leukocytes (or other nucleated cells) and confirmed with genetic testing with the finding of biallelic mutations in the *GBA1* gene.^63^^,^^64^ Fever in patients with Gaucher disease is generally associated with bone manifestations, especially during so-called “bone crises”, which are characterized by severe pain, edema, and often fever, even in the absence of acute radiological changes.^66^ These crises are recurrent and represent one of the main causes of fever in type 1 Gaucher disease, which is the most prevalent form and does not present neurological involvement. Furthermore, infiltration of the mononuclear phagocytic system by engorged macrophages (Gaucher cells) can contribute to systemic inflammatory conditions, favoring febrile episodes. Another relevant aspect is the predisposition to infections, resulting from the immune dysfunction observed in Gaucher disease, especially in untreated patients. Glucocerebrosidase deficiency compromises the microbicidal capacity of monocytes/macrophages, increasing the risk of infections, such as tuberculosis, which can manifest with persistent fever.^67^ In these cases, fever may be secondary to opportunistic infection and tends to regress with specific treatment and the institution of enzyme replacement therapy.

One may also consider the diagnosis of subacute thyroiditis as a rare cause of FUO or Recurrent Fever, with or without clear signs of thyroiditis.^68^ Other causes include thromboembolic phenomena, cardiac myxoma, thyroid disorders, cholesterol emboli, pheochromocytoma, paraganglioma, drug fever associated with gastroplasty, and hemolytic processes.^12^

#### Occupational fever

Fever can arise from diseases of diverse etiologies, including some with occupational origins. Examples include metal fume fever and polymer fume fever, both caused by inhalation exposures. Metal fume fever typically manifests nonspecific symptoms such as influenza-like illness, fever, chills, arthralgia, myalgia, headache, and malaise. Symptom onset generally occurs 4- to 10-hours following exposure to metal-containing fumes. Although metal fume fever is generally benign and self-limited, severe cases have been documented. In individuals with continuous exposure over a workweek, tachyphylaxis may develop, characterized by symptom attenuation during the workweek and symptom recurrence following a period of absence from exposure, such as over the weekend. Polymer fume fever is clinically indistinguishable from metal fume fever, making a thorough exposure history essential for differentiation.^69^

#### Factitious fever

Factitious fever represents a heterogeneous clinical entity characterized by the intentional production or feigning of fever, either self-induced or fraudulent. Diagnosis should be considered when patients present with prolonged fever in the absence of corroborating clinical or hematological abnormalities.^70^ Early recognition is critical to avoid unnecessary and costly investigations, which may involve prolonged hospital stays with attendant risks and healthcare expenditures. A retrospective study identified factitious fever in 2.2 % (11/506) of patients initially classified as having fever of unknown origin. Methods of feigning fever included manipulation of thermometers, falsification of records, ingestion of pyrogenic substances, or inoculation with contaminated materials.^71^ Beyond exclusion of genuine organic disease, a comprehensive psychopathological assessment is essential to identify underlying psychiatric or behavioral disorders contributing to this presentation.^72^

## Conclusion

Recurrent fevers present a significant diagnostic challenge, encompassing a wide range of conditions, from infectious and autoimmune diseases to rare genetic disorders such as the inborn errors of immunity. Appropriate management requires a systematic, multifaceted approach, including a detailed history, careful clinical evaluation, and thorough laboratory investigation. Identifying underlying causes can be complex, often requiring advanced complementary tests such as genetic testing, PET/CT imaging, or biopsies, along with a rigorous process of exclusion to rule out more common and severe conditions. Moreover, careful consideration of epidemiological factors is a key component to effectively guide the diagnostic process. Fig. 1 summarizes the proposed diagnostic flowchart.

Limitations of this study are the lack of original Brazilian clinical studies on fever of unknown origin. Access to some diagnostic measures, such as PET scans and biopsies, may not be widespread in the context of healthcare in the Global South. For the sake of brevity, some causes of fever were not addressed.

## Author's contribution

LOM: Data curation; formal analysis; writing-original draft; writing-review & editing. VLPA: Data curation; writing-review & editing. EFP: Writing-review & editing; visualization. MHF: Conceptualization; methodology; data curation; formal analysis; writing-original draft; writing-review & editing; supervision; project administration.

## Declaration of competing interest

The authors declare no conflicts of interest.

## Data Availability

The datasets generated and/or analyzed during the current study are available from the corresponding author upon reasonable request.
